# Delta Lactate (Three-hour Lactate Minus Initial Lactate) Prediction of In-hospital Death in Sepsis Patients

**DOI:** 10.7759/cureus.7863

**Published:** 2020-04-27

**Authors:** Amanda L Webb, Nicholas Kramer, Javier Rosario, Larissa Dub, David Lebowitz, Kendra Amico, Leoh Leon, Tej G Stead, Ariel Vera, Latha Ganti

**Affiliations:** 1 Emergency Medicine, University of Central Florida College of Medicine, Orlando, USA; 2 Emergency Medicine, Brown University, Providence, USA; 3 Emergency Medicine, University of Central Florida College of Medicine/Hospital Corporation of America Graduate Medical Education Consortium of Greater Orlando, Kissimmee, USA; 4 Emergency Medicine, Osceola Regional Medical Center, Kissimmee, USA; 5 Emergency Medicine, Envision Physician Services, Nashville, USA; 6 Emergency Medicine, University of Central Florida College of Medicine/Hospital Corporation of America Graduate Medical Education Consortium of Greater Orlando, Orlando, USA; 7 Emergency Medical Services, Polk County Fire Rescue, Bartow, USA

**Keywords:** sepsis, lactate

## Abstract

This study examines the relationship between serial serum lactate levels and in-hospital mortality in an adult cohort of emergency department patients with severe sepsis or septic shock. Of the 164 patients in the cohort, 130 also got three-hour lactate in addition to the initial one. The median initial lactate was 3.01 (interquartile range [IQR]: 1.71-4.62). The median repeat lactate was 2.58 (IQR: 1.4-3.9). The in-hospital death rate was 23% for men and 29% for women. The delta lactate was significantly higher in women (P=0.0070), driven by a lower initial lactate (P=0.0277). In a multivariate regression model controlled for age and gender, a statistically significant correlation was noted between an increase in the delta lactate and in-hospital death (P=0.0323; R^2^=11.3%). The results of this single-center study suggest that an increase in serum lactic acid is significantly associated with higher in-hospital death.

## Introduction

Severe sepsis and septic shock continue to be a major burden in society. Estimates for the number of cases of severe sepsis in 2009 were as high as 3.1 million with estimated 750,000 deaths [1,2]. Septicemia was listed as the sixth most common primary admitting diagnosis and the most expensive, with almost $15.4 billion spent in aggregate hospital costs [3].

Although mortality in recent years has trended downward, the incidence of severe sepsis has risen in recent years. It has been suggested that the increasing number of severe sepsis may be due to the “Will Rogers phenomenon” or stage migration, where increased awareness coupled with a lower threshold for testing resulted in diagnosing less sick patients, increasing the incidence, and therefore decreasing the overall mortality rate [4,5]. Some argue that the increase may be due to increases in the average age of the population, number of people living with chronic conditions, and number of people undergoing treatments that negatively affect the immune system, such as chemotherapy and organ transplants. Decreased case fatality has also been attributed to advances in critical care [6].

As outcomes are improved with earlier initiation of treatment, much effort has been put forth to identify markers differentiating early sepsis from noninfectious systemic inflammatory response syndrome (SIRS) and to aid in prognostication. C-reactive protein elevation has been a traditional marker of inflammation, but it is not specific to infection and is not generally elevated until six to eight hours after introduction of a pathogen. Lactic acid, procalcitonin, presepsin, CD64, soluble urokinase-type plasminogen activator receptor (suPAR), and soluble triggering receptor expressed on myeloid cells 1 (TREM-1) are other biomarkers currently being investigated [7-9].

We wanted to examine the relationship between the delta lactate (three-hour lactate minus the initial lactate) and in-hospital death in patients with severe sepsis and septic shock. We hypothesized that a positively trending lactic acid at three hours despite adequate resuscitation in patients diagnosed with severe sepsis or septic shock would result in a greater in-hospital mortality rate.

## Materials and methods

We assessed an observational cohort consisting of all patients diagnosed with sepsis in the emergency department (ED) who were coded as severe sepsis or septic shock during their hospital stay. Our study was conducted at an urban ED, with 75,000 visits per year, between July 2016 and March 2017. Initial lactic acid, three-hour lactic acid, and in-hospital mortality were abstracted onto pre-designed data collection sheets by abstractors blinded to outcome. The data sheet included demographic, laboratory, and hospital outcome variables.

The inclusion criteria consisted of adults over the age of 18, ED diagnosis of severe sepsis, or septic shock by two or more of the following: temperature of more than 38°C (100.4°F) or less than 36°C (96.8°F), heart rate greater than 90 beats per minute, respiratory rate greater than 20 breaths per minute, or abnormal white blood cell count ( >12,000/µL or <4,000/µL or >10% immature [band] forms) and associated organ dysfunction, hypoperfusion, or hypotension (systolic blood pressure < 90 mm Hg or a rapid decrease from baseline), or persistent hypotension and perfusion abnormalities despite adequate fluid resuscitation.

Exclusion criteria consisted of anyone who developed sepsis after the ED visit (during hospitalization), patients transferred from another facility, anyone less than 18 years of age, and patients who did not have an initial or three-hour lactic level recorded during their hospital stay.

 A multivariate regression model was performed to decipher whether delta lactate was a predictor of an in-hospital death. Statistical analyses were conducted in JMP 12.0 for Mac (SAS Institute Inc., Cary, NC, USA). These results were presented in an abstract form at the American College of Emergency Physicians Research forum [10].

## Results

A total of 164 patients were determined to have sepsis or septic shock first noted in the ED. Of the patients, 74 (45%) met the criteria for severe sepsis, whereas 90 (55%) were classified as septic shock. Median age was 72 years old, with an interquartile range (IQR) of 60-82. The cohort consisted of 164 patients who had an initial lactate drawn, 130 of which also had a three-hour lactate drawn.

**Figure 1:**
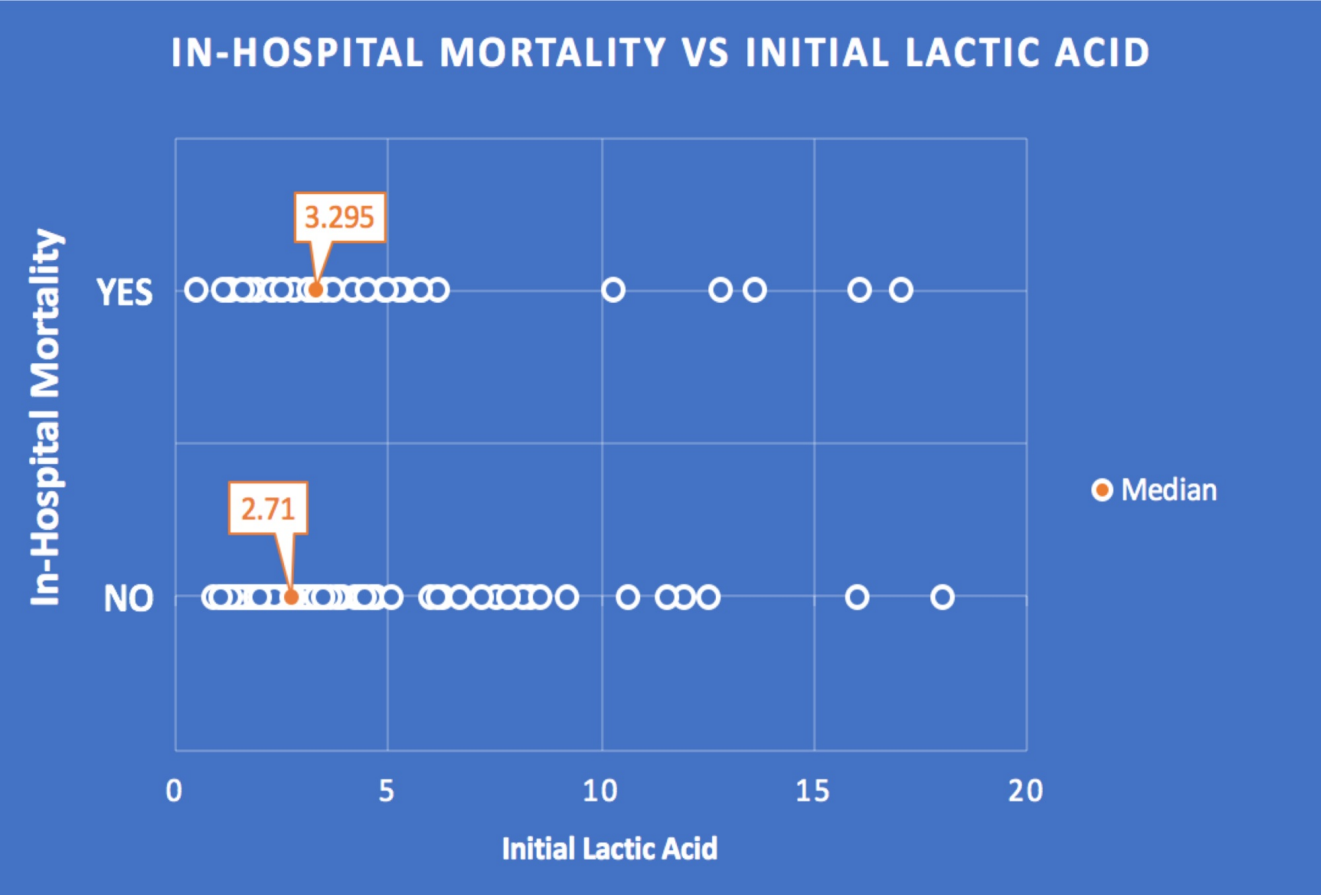
In-hospital mortality vs. initial lactic acid

The median initial lactate was 3.01, with an IQR of 1.71 to 4.62 and a range of 0.49 to 18.04. The median initial lactate of those who survived was 2.71 compared with 3.29 for those who died (Figure 1).

**Figure 2:**
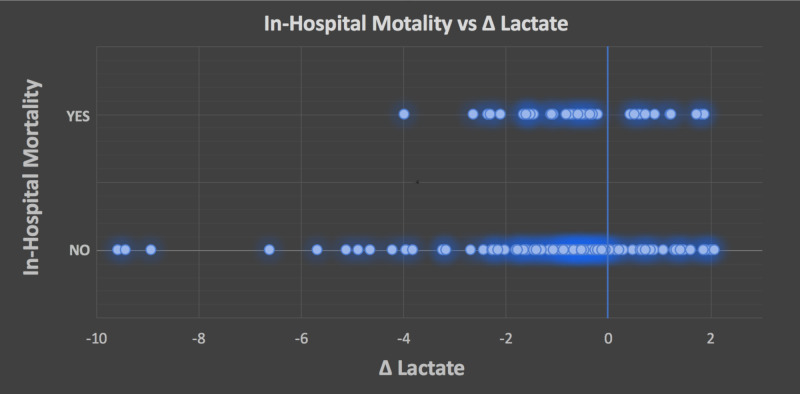
In-hospital mortality vs. delta lactate

The median repeat lactate was 2.58, with an IQR of 1.4 to 3.9 and a range of 0.4 to 28.7. Of the cohort, 26% died during their hospital stay. The in-hospital death rate was 23% for men and 29% for women. Of the 34 patients with a positive delta lactic acid, 12 died in hospital (35.29%), whereas 22 of the 96 (22.92%) patients with a negative delta lactic acid died (Figure 2).

Multivariate regression model controlled for age and gender demonstrated a statistically significant correlation between an increase in the delta lactate and in-hospital death (p=0.0323; R^2^=11.3).

## Discussion

Every septic patient is on a spectrum ranging from sepsis to septic shock. Where they are on that spectrum has a significant impact on expected outcomes, mortality estimates ranging from 10% in sepsis upward to 40% in septic shock [3]. Early identification of sepsis patients at high risk of early death could direct higher level care earlier to those who may benefit the most. This has sparked a significant amount of research to find biomarkers and develop clinical prediction tools to risk stratify patients presenting to the ED [9]. In one study, patients who were admitted directly to the intensive care unit (ICU) from the ED had better outcomes than patients who were transferred to the ICU after deterioration on the general medical floor [2].

**Figure 3:**
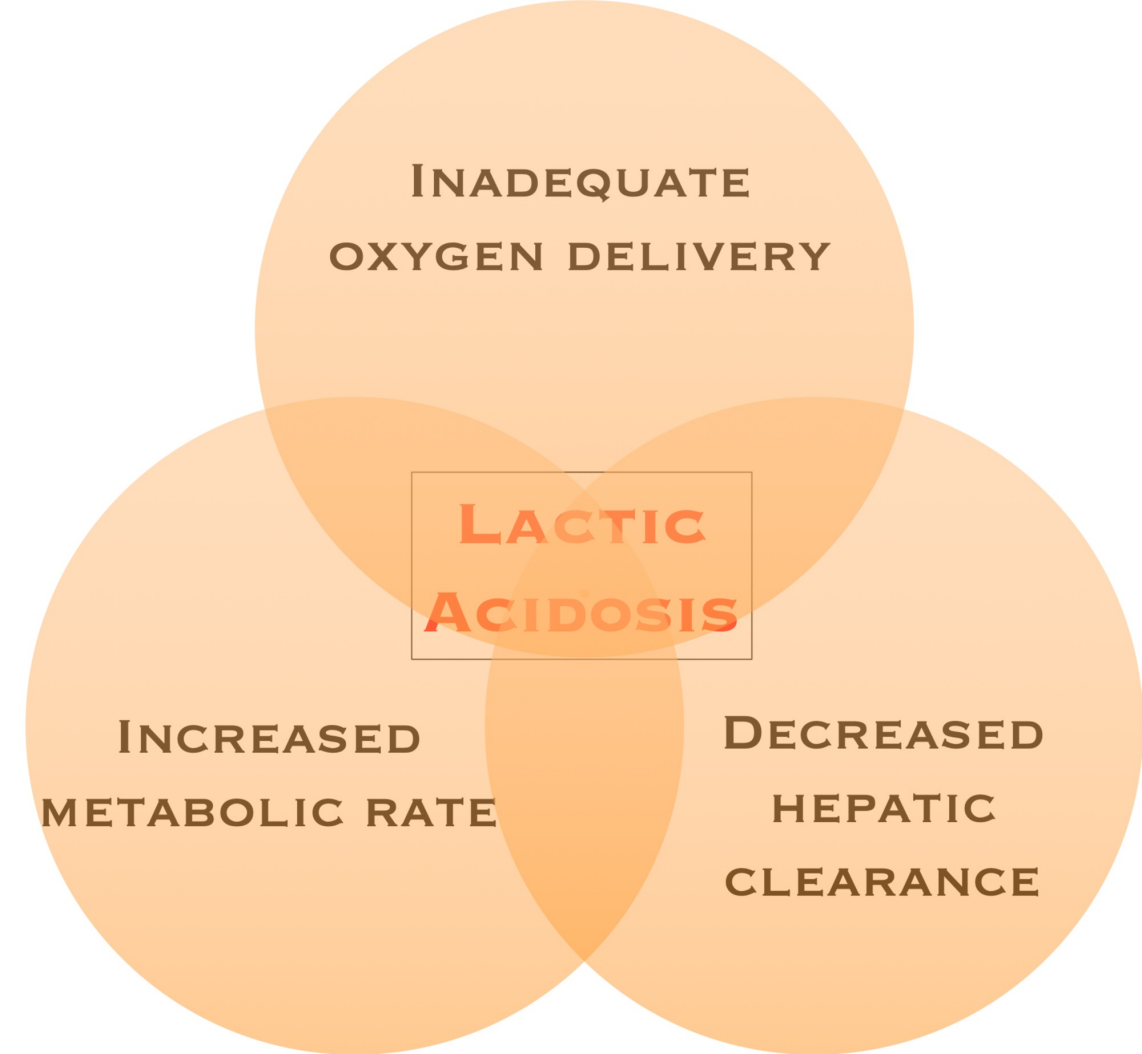
Lactic acidosis triad

Lactate, although not specific to sepsis, is the most commonly used biomarker to assess illness severity and response to treatment. Patients presenting with severe sepsis or septic shock often have an elevated lactate level (Figure 3) [8].

Lactate was traditionally thought to correlate with the level of tissue hypoxia and inadequate whole body oxygen delivery and has been labeled as type A hyperlactatemia. Type B hyperlactatemia occurs in the setting of inadequate tissue perfusion through increased production or impaired clearance. Examples include excess beta-adrenergic stimulation, impaired tissue oxygen extraction, and mitochondrial dysfunction leading to anaerobic metabolism. Clearance can be significantly impaired in hepatic dysfunction as the liver is the primary metabolizer of lactate [11-13].

High initial lactate levels, failure to normalize lactate levels, and early positive blood cultures in sepsis patients presenting to the ED are associated with poorer outcomes and high in-hospital mortality [8,9,12,14]. The most common cutoff values for hyperlactatemia were 2 mmol/L for a low threshold and 4 mmol/L often for protocolized resuscitation [15]. Both cutoffs have been associated with increased mortality at 24 hours and 28 days [8,9,12,14]. However, normal and lactate levels ≤ 2 have not been shown to rule out future deterioration to septic shock and death [16].

Previous research has shown mixed results regarding lactate-guided resuscitation [12,17,18]. Several studies evaluate lactate clearance defined as [(lactate initial - lactate repeat)/lactate initial] * 100%, where ≥ 10% is used as a cutoff [9,19,20]. Failure to normalize lactate is strongly associated with early death [9]. One study found higher in-hospital mortality in sepsis patients with a repeat lactate drawn within six hours that was ≥4 mmol/L, with less than 20% relative reduction. However, a less than 10% reduction was not significant, whereas it was in other studies that did not use 4 mmol/L as a cutoff [20]. Another study also used a delta lactate defined as (lactate initial - lactate repeat). They did not find a significant difference in delta lactate, whereas we found that a positive delta lactate was significantly associated with mortality [9].

## Conclusions

An increase in the three-hour lactic acid as compared with the initial acid was significantly associated with higher in-hospital death in our cohort of severe sepsis and septic shock patients who presented to the ED. This information may be utilized to accelerate a higher level of care for these patients.

## References

[1] Gaieski Gaieski, DF DF, Edwards M, Kallan MJ, Carr BG (2013). Benchmarking the incidence and mortality of severe sepsis in the United States. Crit Care Med.

[2] Whittaker SA, Fuchs BD, Gaieski DF (2015). Epidemiology and outcomes in patients with severe sepsis admitted to the hospital wards. J Crit Care.

[3] Elixhauser A, Friedman B, Stranges E (2020). Septicemia in U.S. hospitals, 2009. Healthcare Cost and Utilization Project (HCUP) Statistical Briefs.

[4] Stoller J, Halpin L, Weis M (2016). Epidemiology of severe sepsis: 2008-2012. J Crit Care.

[5] Iwashyna TJ, Angus DC (2014). Declining case fatality rates for severe sepsis: good data bring good news with ambiguous implications. JAMA.

[6] Mayr FB, Yende S, Angus DC (2014). Epidemiology of severe sepsis. Virulence.

[7] Larsen FF, Petersen JA (2017). Novel biomarkers for sepsis: a narrative review. Eur J Intern Med.

[8] Fan S, Miller NS, Lee J, Remick DG (2016;460). Diagnosing sepsis-the role of laboratory medicine. Clin Chim Acta.

[9] Javed A, Guirgis FW, Sterling SA (2017). Clinical predictors of early death from sepsis. J Crit Care.

[10] Kramer N, Leon L, Rosario J (2020). 222 delta lactate (3-hour lactate minus initial lactate) predicts in-hospital death in sepsis patients. Ann Emerg Med.

[11] Suetrong B, Walley K (2016). Lactic acidosis in sepsis: it’s not all anaerobic: implications for diagnosis and management. Chest.

[12] Rhodes A, Evans LE, Alhazzani W (2017). Surviving sepsis campaign: international guidelines for management of sepsis and septic shock: 2016. Intensive Care Med.

[13] Van den Nouland DP, Brouwers MC, Stassen PM (2020). Prognostic value of plasma lactate levels in a retrospective cohort presenting at a university hospital emergency department. BMJ Open.

[14] Mikkelsen ME, Miltiades AN, Gaieski DF (2009). Serum lactate is associated with mortality in severe sepsis independent of organ failure and shock. Crit Care Med.

[15] Thomas-Rueddel DO, Poindinger B, Weiss M (2015). Hyperlactatemia is an independent predictor of mortality and denotes distinct subtypes of severe sepsis and septic shock. J Crit Care.

[16] Fernando SM, Barnaby DP, Herry CL (2018). Helpful only when elevated: initial serum lactate in stable emergency department patients with sepsis is specific, but not sensitive for future deterioration. J Emerg Med.

[17] Gu WJ, Zhang Z, Bakker J (2015). Early lactate clearance-guided therapy in patients with sepsis: a meta-analysis with trial sequential analysis of randomized controlled trials. Intensive Care Med.

[18] Simpson SQ, Gaines M, Hussein Y, Badgett RG (2016). Early goal-directed therapy for severe sepsis and septic shock: a living systematic review. J Crit Care.

[19] Lee SM, Kim SE, Kim EB, et at (2020). Lactate clearance and vasopressor seem to be predictors for mortality in severe sepsis patients with lactate acidosis supplementing sodium bicarbonate: a retrospective analysis. PLoS ONE.

[20] Lokhandwala S, Andersen LW, Nair S (2017). Absolute lactate value vs relative reduction as a predictor of mortality in severe sepsis and septic shock. J Crit Care.

